# Distribution and Variation Characteristics of Branched Glycerol Dialkyl Glycerol Tetraethers (BrGDGTs) in Sediment Cores Along the Nearshore-to-Offshore Gradient of the East China Sea and Their Correlation with Microbial Community Diversity

**DOI:** 10.3390/biology14081077

**Published:** 2025-08-18

**Authors:** Ting Zeng, Cheng Liu, Qunhui Yang, Jingyuan Zhao, Fuwu Ji

**Affiliations:** 1 State Key Laboratory of Marine Geology, Tongji University, Shanghai 200092, China; zengt_1214@163.com (T.Z.); liucheng96@tongji.edu.cn (C.L.); zhaojingyuan@tongji.edu.cn (J.Z.); 2 Project Management Office of China National Scientific Seafloor Observatory, Tongji University, Shanghai 200092, China; 3 Laoshan Laboratory, Qingdao 266237, China

**Keywords:** brGDGTs, potential biological producers, bacterial community diversity, depth-dependent distribution pattens, nearshore-to-offshore

## Abstract

Branched glycerol dialkyl glycerol tetraethers (brGDGTs) are promising molecular biomarkers widely applied in paleoenvironmental reconstructions, including temperature and pH. However, knowledge of the microorganisms responsible for brGDGT production in marine environments remains limited, which constrains the further development and application of brGDGT-based proxies for reconstructing past marine conditions. In this study, both ‘living’ intact polar lipid-derived brGDGTs (IPL-brGDGTs) and ‘fossil’ core brGDGTs (CL-brGDGTs), together with bacterial community compositions, were analysed in multiple sediment cores collected along a nearshore-to-offshore transect in the East China Sea (ECS). The potential correlations between brGDGT distributions and bacterial community compositions at varying sediment depths across an environmental gradient were also explored. Results revealed that IPL-brGDGTs were predominantly biosynthesised in situ, whereas CL-brGDGTs reflected a mixture of marine autochthonous production and terrestrial inputs. Potential brGDGT-producing bacteria in nearshore environments were primarily composed of chemolithoautotrophic taxa (e.g., Gammaproteobacteria and Dehalococcoidia) and chemoheterotrophic taxa (e.g., Alphaproteobacteria, Bacilli, and Actinobacteria). In contrast, offshore regions were dominated by chemoheterotrophic hypoxic bacteria (e.g., Anaerolineae and Phycisphaerae) and facultatively anaerobic chemolithoautotrophic bacteria (e.g., Gammaproteobacteria and Desulfobacteria). A significant difference in bacterial community composition and IPL-brGDGT distribution was observed at a depth of 17 cm, likely due to physical disturbance in near-surface sediments, such as wave action, tidal forces, and storm events. Variance partitioning analysis (VPA) revealed that the bacterial community composition alone accounted for 14.1% of the variation in IPL-brGDGTs and 6.5% in CL-brGDGTs, further suggesting that the distribution of brGDGTs is primarily influenced by the composition of the bacterial community in the nearshore-to-offshore sedimentary ecosystems of the ECS. These findings regarding the potential biosynthesis of brGDGTs in coastal habitats advance our understanding of the microbial mechanisms that regulate brGDGT distribution in marine ecosystems. Moreover, they emphasise the importance of considering physical disturbance effects when interpreting sedimentary brGDGT records for paleoenvironmental reconstructions in marginal seas, such as the ECS.

## Simple Summary

Branched glycerol dialkyl glycerol tetraethers (brGDGTs) are promising molecular biomarkers widely applied in paleoenvironmental reconstruction. However, their biological origins within marine ecosystems remain poorly understood. In this study, both ‘living’ intact polar lipid-derived brGDGTs (IPL-brGDGTs) and ‘fossil’ core brGDGTs (CL-brGDGTs), together with bacterial community compositions, were analysed across multiple sediment cores collected along a nearshore-to-offshore gradient in the East China Sea (ECS). The results suggest that Gammaproteobacteria, Dehalococcoidia, Alphaproteobacteria, Bacilli, and Actinobacteria are the primary potential producers of brGDGTs in nearshore environments. In contrast, Anaerolineae, Phycisphaerae, and Desulfobacteria dominate as likely producers in offshore regions. The distribution of brGDGTs is primarily shaped by variations in bacterial community composition. Furthermore, the vertical distribution profiles of both bacterial communities and IPL-brGDGTs—believed to be predominantly synthesised in situ—indicate that physical disturbance processes, such as wave action, tidal forces, and storm events, significantly influence the distributions of bacterial communities and IPL-brGDGTs in near-surface sediments. This study provides new insights into the biological sources of brGDGTs in marine environments. It highlights the importance of considering physical disturbance effects when interpreting sedimentary brGDGT records for paleoenvironmental reconstructions in marginal seas, such as the ECS.

## 1. Introduction

Branched glycerol dialkyl glycerol tetraethers (brGDGTs) are a group of bacterial membrane-spanning lipids characterized by structural features, including 4–6 methyl branches (with outer methyl branches at the C_5_, C_6_, or C_7_ position), 0–2 cyclopentane moie-ties embedded within the carbon chain, and ether linkages with glycerol molecules [1,2,3]. These compounds have been widely detected in various environmental matrices, including soils, peats, and sediments from rivers, lakes, and marine environments [4]. In living cells, brGDGTs exist as intact polar lipids (IPLs). However, following cell death, the polar head groups, such as glycosidic or phosphate moieties, are typically hydrolyzed rapidly (usually within days), resulting in the release of core lipids (CLs) into the surrounding environment [5,6]. Thus, intact polar lipid-derived brGDGTs (IPL-brGDGTs) can serve as tracers for living bacterial biomass [7]. Notably, the degradation dynamics of IPL ethers are influenced by the type of head group, with glycosidic head groups degrading more slowly than phosphate ones [8,9]. As a result, sedimentary IPL-brGDGTs may also contain remnants of dead bacterial cells, representing partially ‘fossilized’ material [10,11,12].

Owing to their remarkable preservation potential (dating back to Mesozoic strata [13]) and high sensitivity to environmental changes, CL-brGDGTs have become the foundation for commonly used proxies of air temperature and soil pH [14,15]. Initially, brGDGTs were thought to be produced solely in soils and peats [1,16]. Their presence in marine sediments was ascribed to exogenous sources like atmospheric dust or riverine input [17]. Consequently, CL-brGDGTs in marginal marine sediment cores have been used to reconstruct past climatic conditions on adjacent continents [18,19]. Nevertheless, an increasing number of studies suggest that brGDGTs can also be produced in the ocean and may serve as indicators of marine temperature [20,21,22], pH [23], and oxygen-related environmental factors [24]. This scenario complicates the application and interpretation of CL-brGDGTs in marginal sea sedimentary records. In contrast, the detection of IPL-brGDGTs in marine environments enables the identification of autochthonous brGDGTs, offering valuable insights into their characteristics in various ecological settings [11,23,25,26].

The microbial producers of brGDGTs were previously hypothesized to belong to Acidobacteriota, which are commonly found in soils [27]. Subsequent studies on cultured representatives have confirmed this hypothesis [28,29,30]. However, the diversity of brGDGTs detected in environmental samples is much greater than that found in cultured Acidobacteria. Moreover, both genetic studies [31,32,33] and field experiments [22,34,35] have revealed a broader range of potential brGDGT-producing organisms. Variations in membrane lipid compositions among different bacterial taxa [28,29] suggest that environmental changes can drive shifts in bacterial community structure, which may, in turn, significantly alter brGDGT distributions [36,37,38]. These findings, together with the homeoviscous adaptation mechanism—whereby membrane lipids are regulated in response to environmental conditions [30,39]—help explain the strong correlations between brGDGT distributions and environmental variables [40,41]. Despite these advances, the identities of brGDGT-producing microbes in marine habitats remain largely unknown, and our understanding of their influence on brGDGT composition and distribution is still limited.

The East China Sea (ECS) is a typical river-dominated marginal sea, where the sedimentary brGDGT pool reflects both terrestrial input and marine autochthonous contribution [42,43,44]. Previous studies have primarily focused on identifying the sources of CL-brGDGTs in suspended particulate matter (SPM) [45] and surface sediments [42,43,44,46,47,48]. These investigations reveal that CL-brGDGTs in the nearshore ECS are predominantly of terrestrial origin, characterized by a low degree of cyclization [42,43,44,45,46,48]. In contrast, the higher cyclization values detected in the offshore regions suggest an increasing contribution from marine autochthonous sources [42,43,44,45,46,47]. However, recent analyses of both IPL-brGDGTs and CL-brGDGTs in ECS surface sediments indicate that nearshore brGDGTs may not accurately represent significant terrestrial inputs, as the cyclization degree of in situ autochthonous IPL-brGDGTs increases offshore, which is consistent with the trends observed for CL-brGDGTs. This suggests that nearshore CL-brGDGTs in the ECS may also have a substantial marine authigenic origin [23,49].

Recently, brGDGTs have been proposed to reconstruct seawater temperature or pH, based on the understanding that brGDGTs in ECS surface sediments are primarily derived from planktonic bacteria [22,23]. However, Chen et al. pointed out that alterations in the structure of benthic bacterial communities could be the crucial factor influencing the distribution of brGDGTs in ECS surface sediments [35]. Moreover, although the methylation level of 6-methyl bicyclopentane brGDGTs—mainly produced by marine bacteria—in surface sediments from the ECS and Yellow Sea shows a significant correlation with the annual mean sea surface temperature (SST) in the region, this correlation is regulated by bacterial communities in subsurface seawater and benthic sediments [22].

Overall, previous studies on brGDGTs in the ECS have predominantly focused on SPM and surface sediments. In contrast, relatively little attention has been paid to IPL-brGDGTs in sediment cores. Moreover, the potential link between variations in benthic bacterial communities and brGDGT distributions across nearshore-to-offshore sedimentary environments in the ECS has received scant attention. This knowledge gap hinders a comprehensive understanding of the sources of brGDGTs and, to some extent, compromises the accuracy of paleoenvironmental reconstructions based on brGDGT distributions in marine settings.

In this study, we analyzed both IPL- and CL-brGDGTs, together with microbial diversity, in multiple sediment cores collected along a nearshore-to-offshore transect in the ECS. Our objectives were to identify potential brGDGT-producing microorganisms and evaluate how bacterial community composition influences brGDGT distribution across the shallow ECS shelf. The results are expected to enhance our understanding of the in situ biosynthesis of brGDGTs in marginal seas and the complex factors governing their spatial distribution, thereby improving the reliability of paleo-oceanic environment reconstructions using brGDGT proxies.

## 2. Materials and Methods

### 2.1. Study Area and Sample Collection

The sediments on the ECS shelf primarily derive from river discharge, island input, and coastal erosion [50]. The Yangtze River delivers 0.34–0.5 × 10^9^ tons of sediment annually to the ECS [51], serving as the principal source of ECS shelf sediments. The distribution of terrestrial material in this region is primarily influenced by the Yangtze River Diluted Water (YDW), the Zhejiang-Fujian Coastal Current (ZFCC), the Taiwan Warm Current (TWC), and the East Asian Monsoon [52]. In summer, most terrestrial particles are transported eastward and northeastward, accumulating in the Yangtze River Estuary (YRE) and adjacent areas, with a limited contribution to sediments on the ECS shelf. In winter, terrestrial particles entering the estuary are predominantly carried southward along the coast by the YDW and ZFCC. The eastward dispersion of fine-grained terrestrial materials is obstructed by the northeastward TWC on the middle shelf, resulting in the formation of muddy zones on the ECS inner shelf [52,53].

Tides and currents are crucial physical processes affecting sediment distribution in the ECS. The region experiences predominantly semidiurnal tides, with tidal ranges increasing from 1–2 m on the eastern coast to 2–6 m on the western coast, and peaking at 8.9 m in the Qiantang River estuary. Tidal current velocities reach 200 cm/s at the YRE, declining to 5–10 cm/s on the open shelf [54]. Additionally, summer typhoons and strong winter winds induce intense hydrodynamic disturbances in ECS waters [55,56,57]. Strong tidal currents and wind-generated waves frequently resuspend sediments on the ECS shelf, particularly in coastal zones [58].

**Figure 1:**
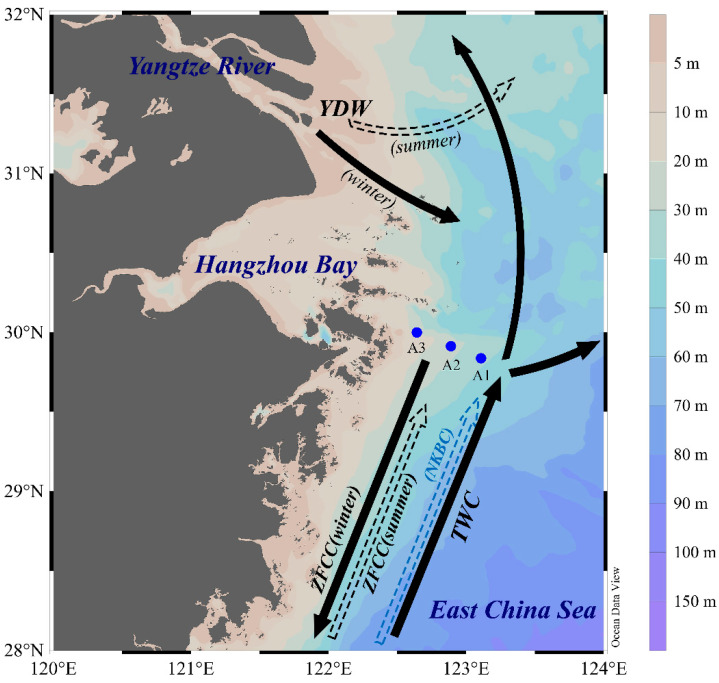
Geographical locations of core sediment sampling sites (A1, A2, A3) and major ocean currents in the study region. YDW: Yangtze River Diluted Water; ZFCC: Zhejiang-Fujian Coastal Current; TWC: Taiwan Warm Current; NKBC: Nearshore Kuroshio Branch Current. Solid arrows depict the winter flow directions of the YDW and ZFCC, whereas dashed arrows illustrate their summer flow directions. Modified from [59,60,61].

The sampling area of this study is situated in the ECS, adjacent to the Zhoushan Islands in Zhejiang Province. In August 2021, three sediment cores were retrieved using a multi-core sampler with a diameter of 10 cm (Figure 1, Table 1). The cores were sliced at 2 cm intervals on the deck. Each sediment sample was individually wrapped in aluminium foil preheated to 450 °C, placed into sealed plastic bags, labelled, and stored at temperatures below −20 °C for further analysis.

### 2.2. Analysis of the Total Organic Carbon (TOC) and Its Stable Isotope Ratios

Sediment samples were analyzed for TOC, total nitrogen (TN), and organic carbon stable isotope ratios (δ^13^C) according to the methods previously detailed in [62,63]. For each sample, 2–3 g of freeze-dried sediment was decarbonized using 4 N HCl, followed by washing to neutrality with ultrapure water, and then freeze-dried. The samples were placed in tin boats, and the organic elemental analyser (Vario EL Cube, Elementar, Hanau, Germany) was employed to measure the TOC and TN levels. The standard deviations (SDs) of six replicate measurements for the same sample were ±0.02% for TOC and ±0.01% for TN. Stable carbon isotope ratios were measured by an elemental analyser (Flash EA1112, Thermo Fisher, Waltham, MA, USA) coupled with an isotope ratio mass spectrometer (DELTAplus XL, Thermo Fisher, Waltham, MA, USA). Replicate analyses of one sample (*n* = 6) yielded a precision of ± 0.06‰. The δ^13^C results were reported relative to the V-PDB international standard.

### 2.3. Lipid Extraction and IPL/CL Separation

Lipid extraction was performed using a modified Bligh-Dyer method [7]. In brief, approximately 7 g of freeze-dried sediment was sonicated three times with a solvent mixture comprising methanol (MeOH), dichloromethane (DCM), and phosphate buffer (PB, pH 7.4; 2:1:0.8, *v*/*v*/*v*), followed by three extraction processes using MeOH, DCM, and trichloroacetic acid buffer (TCA, 50 g/L, pH 2; 2:1:0.8, *v*/*v*/*v*). DCM and ultrapure water were added to the extract to provide a MeOH:DCM:H_2_O ratio of 1:1:0.9 for phase separation. The lipid-containing DCM solution was then transferred into a new specimen bottle, and the remaining solution was extracted 2–3 more times with DCM. All DCM solutions with the lipid extract were concentrated by rotary evaporation. Then, water and contaminants were removed via an anhydrous sodium sulphate column. Finally, the purified total lipid extract (TLE) was acquired through gentle nitrogen blow-drying. In the internal standard recovery experiment, the lipid recovery rates exceeded 85%.

CL and IPL were separated via column chromatography [64]. Briefly, the TLE was redissolved in a hexane: ethyl acetate mixture (1:1, *v*/*v*) and then separated on an activated silica gel column. The CL fraction was eluted with approximately 14 mL of n-hexane/ethyl acetate (1:1, *v*/*v*). Subsequently, the IPL fraction was eluted using approximately 6 mL of MeOH. 50 μL of the internal standard C_46_ GDGT (20 ng/µL) was added to both the CL and IPL fractions [65]. The IPL fraction was further divided into two aliquots. One (IPL-1) was hydrolyzed with MeOH/HCl (95:5, *v*/*v*) at 70 °C for 3 h to remove the headgroups. The other aliquot (IPL-2) was used to quantify the residual CL-GDGT. The amount remaining from IPL-1 was subtracted from the total IPL-GDGTs and added to the CL-GDGTs fraction (the residual fraction comprised less than 3% of the total IPL-GDGTs). The CL, IPL-1 (hydrolyzed into CL-GDGT), and IPL-2 fractions were evaporated and subsequently redissolved in a hexane: isopropanol mixture (99:1, *v*/*v*). The solution was filtered through a 0.45 μm PTFE filter, dried with nitrogen, and stored at −20 °C until analysis.

GDGTs were analyzed on high-performance liquid chromatography-atmospheric pressure chemical ionisation mass spectrometry (HPLC-APCI-MS). Two silica liquid chromatography columns in sequence (2.1 mm × 150 mm, 3 μm; Alltech, Deerfield, IL, USA) were applied at 40 °C to separate 5- and 6-methyl brGDGTs. Mobile phases A and B were hexane and a 90% hexane/10% isopropanol mixture, respectively. The elution gradient was as outlined below: 0–25 min: 82% A and 18% B; 25–60 min: linear increase to 35% B; 60–80 min: linear increase to 100% B, maintained for 10 min; 20 min equilibration [66]. GDGTs were detected with single ion monitoring (SIM) at *m*/*z* 1050, 1048, 1046, 1036, 1034, 1032, 1022, 1020, 1018, and 744. The quantification of GDGTs was achieved by comparing the peak area of each component with that of the C_46_ GDGT internal standard. The SD of a replicate analysis was 5.0% of the concentration for each compound. The method detection limits for each GDGT were 0.01–0.1 ng/g dw, estimated as the content yielding a peak with a signal-to-noise ratio of 3.

### 2.4. Proxy Calculations

%0 Ring, %1 Ring, and %2 Rings, respectively represent the relative abundances of brGDGTs with 0, 1, and 2 cyclopentane rings. The calculation formulas are as follows:

(1) $$\%0\;Ring=\frac{Ia+IIa+IIa^{\prime }+IIIa+IIIa^{\prime }}{\Sigma brGDGTs}\times 100\%$$

(2) $$\%1\;Ring=\frac{Ib+IIb+IIb^{\prime }+IIIb+IIIb^{\prime }}{\Sigma brGDGTs}\times 100\%$$

(3) $$\%2\;Rings=\frac{Ic+IIc+IIc^{\prime }+IIIc+IIIc^{\prime }}{\Sigma brGDGTs}\times 100\%$$

where the Roman numerals I, II, and III represent the tetramethylated, pentamethylated, and hexamethylated brGDGTs series, respectively; the subsequent a, b, and c denote brGDGTs containing 0, 1, and 2 cyclopentane rings, respectively; an apostrophe indicates an external methyl at C_6_, and no apostrophe indicates it at C_5_.

The ${\#Rings}_{tetra}$ index is defined as the weighted average number of cyclopentane moieties in tetramethylated brGDGTs, with higher values indicating a higher degree of cyclization. The calculation formula follows Sinninghe Damsté et al. [67]:

(4) $${\#Rings}_{tetra}=\frac{Ib+2\times Ic}{Ia+Ib+Ic}$$

The methylation of branched tetraethers based only on the 5-methyl isomers (defined as ${{MBT}^{\prime }}_{5Me}$) is employed to reconstruct the mean annual air temperature (MAT) of terrestrial environments. A higher ${{MBT}^{\prime }}_{5Me}$ value implies lower methylation, corresponding to higher synthesis temperatures. The calculation formulas are as follows [15]:

(5) $${{MBT}^{\prime }}_{5Me}=\frac{Ia+Ib+Ic}{Ia+Ib+Ic+IIa+IIb+IIc+IIIa}$$

(6) $$MAT=-8.57+31.45\times {{MBT}^{\prime }}_{5Me}$$

The cyclization of branched tetraethers based only on the 5-methyl isomers (defined as ${CBT}_{5Me}$) can be used to reconstruct soil pH [15]. A lower ${CBT}_{5Me}$ index value indicates a higher degree of cyclization, corresponding to a higher soil pH. The relevant formulas are as follows:

(7) $${CBT}_{5Me}=-log(\frac{Ib+IIb+{IIb}^{\prime }}{Ia+IIa+{IIa}^{\prime }})$$

(8) $$pH=7.84-1.73\times {CBT}_{5Me}$$

### 2.5. DNA Extraction, 16S rRNA Gene Sequencing, and Data Analyses

Whole environmental DNAs were extracted from 30 sediment samples selected at intervals using the DNeasy PowerSoil Kit (Qiagen, Hilden, Germany) following the manufacturer’s instructions. Approximately 0.30 g of each sample was used. The quality of the isolated genomic DNA was evaluated by 1% agarose gel electrophoresis, and the concentration and purity of the DNA were measured using a NanoDrop 2000 (Thermo Scientific Inc., Waltham, MA, USA).

The V3-V4 variable region of the 16S rRNA gene within the isolated DNA was amplified through PCR. The forward primer employed was 515FmodF (5′-GTGCCAGCMGCCGCGGTAA-3′), while the reverse primer was 806RmodR (5′-GGACTACNVGGGTWTCTAAT-3′) [68]. The composition of the PCR reaction mixture and the amplification protocol were executed according to the method described by Walters et al. [68]. The PCR products were separated via 2% agarose gel electrophoresis, purified using the AxyPrep DNA Gel Extraction Kit (Axygen Biosciences, Union City, CA, USA), and quantified with QuantiFluor™-ST (Promega, Madison, WI, USA). Subsequently, the purified and quantified PCR products were sequenced on the MiSeq PE300 platform (Illumina, San Diego, CA, USA) by Majorbio Bio-Pharm Technology Co., Ltd. (Shanghai, China). The raw data were submitted to the NCBI database under the BioProject ID PRJNA1283994.

The raw sequences acquired from Illumina paired-end sequencing were processed in accordance with the QIIME2 standard pipeline [69]. Specifically, Fastp (v0.19.6) [70] was utilized for quality control and filtration. FLASH (v1.2.11; http://www.cbcb.umd.edu/software/flash, accessed on 3 May 2025; [71]) was employed to perform sequence assembly. Operational taxonomic units (OTUs) were clustered at a 97% similarity level by using UPARSE v7.1 software (http://drive5.com/uparse/, accessed on 3 May 2025; [72,73]), after which chimeric sequences were removed. Sequences identified as chloroplasts and mitochondria were excluded from all samples. To mitigate the influence of sequencing depth on subsequent analysis, all samples were rarefied to the minimum sequence count. The RDP classifier [74] (https://github.com/rdpstaff/classifier, accessed on 3 May 2025, version 2.11) was used for OTU taxonomic classification, referring to the Silva 16S rRNA gene database (v138.2) [75]. Only bacterial sequences were retained for further analysis, with a confidence threshold of 70%. The community composition of each sample was evaluated at multiple taxonomic levels. Spearman’s rank correlation was used to calculate the pairwise correlations between the relative abundances of bacterial classes and brGDGTs. The Benjamini–Hochberg (BH/FDR) procedure was adopted for multiple testing correction to control false discovery rates. All analyses were carried out in R (v4.4.3). Significant positive correlations were visualized as co-occurrence networks in Gephi (v0.10.1) [76,77]. To investigate the impact of organic matter content on brGDGTs [78,79], a partial redundancy analysis (partial RDA) was performed using the rdacca.hp (https://cran.r-project.org/web/packages/rdacca.hp/index.html, accessed on 8 May 2025). Additionally, variance partitioning analysis (VPA) was carried out with the vegan package (https://cran.r-project.org/web/packages/vegan/, accessed on 11 May 2025) on all samples with both microbial and organic matter composition data. The aim was to evaluate the influence of bacterial community composition and organic matter concentration on brGDGTs. The parameters used were α = 0.05 (significance level), test power = 0.8 (adequate power to detect significant effects), 1000 permutations (for reliable *p*-value estimation), and 95% CIs (to quantify the uncertainty of the results) [79].

## 3. Results

### 3.1. TON, TN Content and δ^13^C

**Figure 2:**
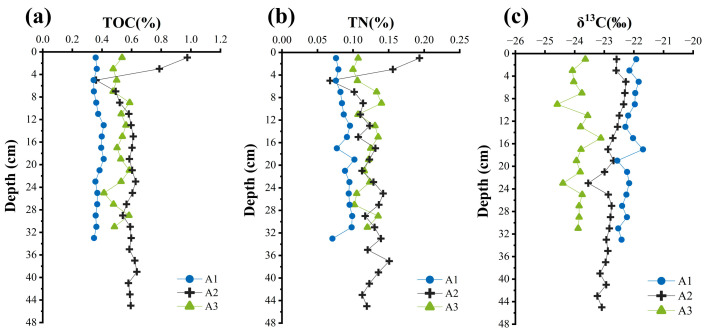
The distributions of organic matter metrics in the sediment cores from sites A1 (blue circles), A2 (black crosses), and A3 (green triangles): TOC (**a**), TN (**b**), and δ^13^C (**c**).

The TOC contents of sediment samples from sites A1, A2, and A3 ranged from 0.34% to 0.41% (mean ± SD: 0.37 ± 0.02%), 0.36% to 0.98% (0.50 ± 0.11%), and 0.41% to 0.59% (0.52 ± 0.05%), respectively, while TN contents ranged from 0.07% to 0.10% (0.09 ± 0.01%), 0.07% to 0.19% (0.13 ± 0.02%), and 0.10% to 0.14% (0.12 ± 0.01%), respectively. Both TOC and TN exhibited a spatial distribution pattern of A2 > A3 > A1 (Figure 2a,b). Vertically, TOC and TN contents at A2 show a significant decrease from the surface to 5 cm depth, followed by a gradual increase in the 5–9 cm interval, and then remain stable (Figure 2a,b). No distinct depth-related patterns were observed at A3 and A1 (Figure 2a,b).

The δ^13^C values of sediment samples from sites A1, A2, and A3 varied between −22.56‰ and −21.69‰ (−22.17 ± 0.23‰), −23.54‰ and −22.27‰ (−22.80 ± 0.30‰), and −24.59‰ and −23.12‰ (−23.86 ± 0.32‰), respectively, with values becoming increasingly heavier from nearshore to offshore. Vertically, δ^13^C at A2 and A1 exhibited a slight decrease from the surface to deeper layers (Figure 2c).

### 3.2. Distributions of IPL- and CL-brGDGTs

Fifteen identified compounds of conventional brGDGTs were observed in the sediment cores from the study area (Table S1 for details). Among them, GDGT-Ia appeared as the predominant component, constituting 29.4–53.9% (40.0 ± 4.9%) of IPL-brGDGTs and 20.1–37.6% (29.2 ± 4.1%) of CL-brGDGTs. The relative abundance of IIa, Ib, and IIa’ was higher in the IPL-brGDGTs fractions, averaging 10.6 ± 1.9%, 12.0 ± 1.8%, and 8.8 ± 1.8%, compared to 15.7 ± 2.2%, 15.1 ± 1.2%, and 9.0 ± 2.9% in the CL-brGDGTs fraction, respectively. Conversely, the mean relative abundances of IIc, IIIb, IIIb’, and IIIc, IIIc’ were extremely low (<1%) in both the CL-brGDGTs and IPL-brGDGTs pools.

**Figure 3:**
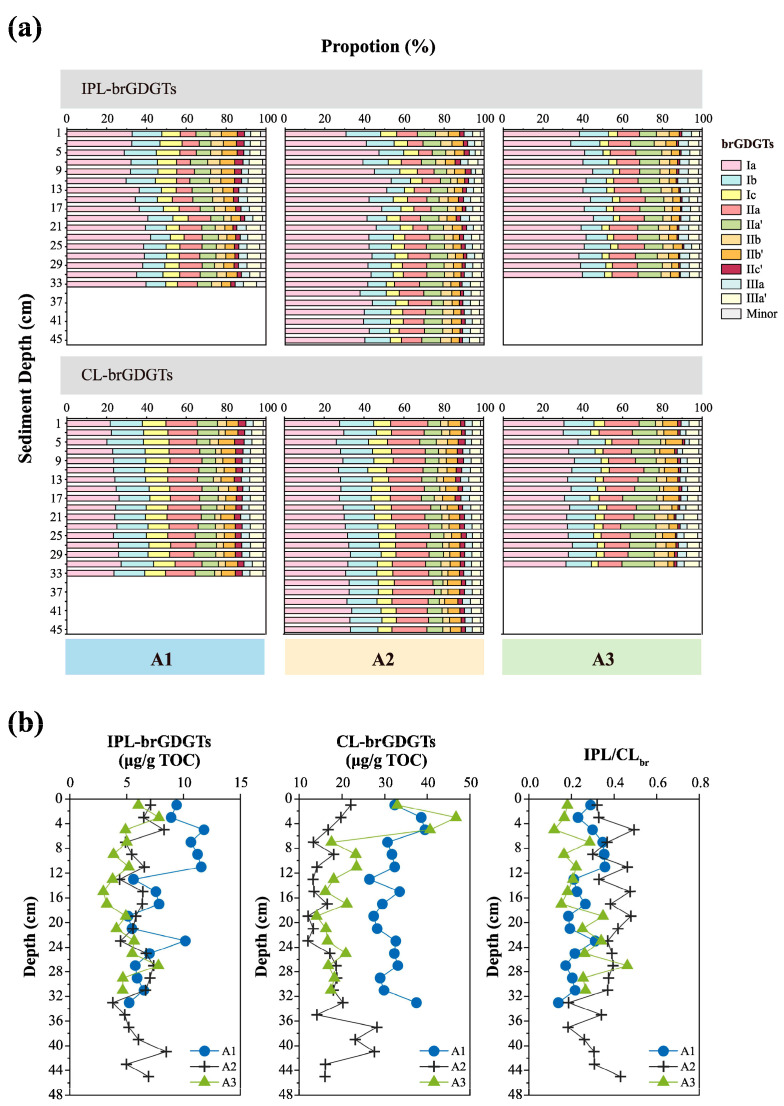
Vertical profiles of the relative abundance, concentrations, and concentration ratios of IPL-derived and core brGDGTs in sediment cores from sites A1, A2, and A3: (**a**) the relative abundance of IPL-derived and core brGDGTs (A1: left, A2: centre, and A3: right); (**b**) concentrations and concentration ratios of IPL-derived and core brGDGTs (A1: blue circles, A2: black crosses, and A3: green triangles).

The concentrations of brGDGTs in IPLs and CLs ranged from 1.58 to 6.91 ng/g dw and 6.03 to 26.13 ng/g dw, respectively (Table S1), with the IPL/CL_br_ concentration ratios varying between 0.12 and 0.49 (Figure 3b, Table S1). To reduce the impacts of factors such as particle size, this study primarily focused on the TOC-normalized concentrations of brGDGTs. At sites A1, A2, and A3, the average concentrations of IPL-brGDGTs were 7.97 ± 2.34 μg/g TOC, 6.03 ± 1.20 μg/g TOC, and 5.00 ± 1.34 μg/g TOC, respectively, increasing from nearshore to offshore. Higher concentrations were detected in the near-surface layers at site A1 and near-bottom layers at sites A1 and A2 (Figure 3b). Regarding CL-brGDGTs, the average concentrations at A1, A2, and A3 were 32.03 ± 3.60 μg/g TOC, 17.58 ± 4.47 μg/g TOC, and 22.51 ± 9.14 μg/g TOC, respectively. Except at site A3, where high concentrations were observed in the near-surface layer, the general distribution pattern presented lower concentrations nearshore and higher concentrations offshore (Figure 3b).

### 3.3. Microbial Community Composition

**Figure 4:**
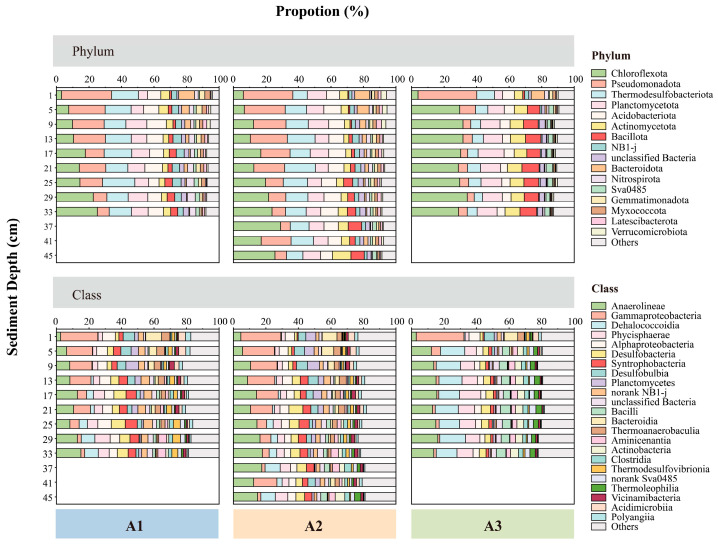
The phylum-level and class-level composition of bacterial communities in sediment cores from sites A1 (**left**), A2 (**centre**), and A3 (**right**). Only bacterial taxa with relative abundances exceeding 1% are incorporated.

Based on the 16S rRNA gene sequencing data, 77 bacterial phyla and 202 classes were identified in the sediment samples of this study. At the phylum level, Chloroflexota (19.2 ± 9.3%) and Pseudomonadota (14.3 ± 8.8%) were the dominant bacterial groups, followed by Thermodesulfobacteriota (12.2 ± 4.0%), Planctomycetota (10.4 ± 2.2%), Acidobacteriota (8.2 ± 2.1%), Actinomycetota (5.7 ± 2.3%), and Bacillota (4.9 ± 3.2%) (Figure 4). The combined relative abundance of the remaining 70 bacterial phyla did not exceed 30% of the total bacterial community, except for sample A3-1 (30.5%). At the class level, the predominant groups were Anaerolineae (11.6 ± 4.2%), followed by Gammaproteobacteria (9.2 ± 7.9%), Dehalococcoidia (6.1 ± 5.5%), Phycisphaerae (5.9 ± 2.9%), Alphaproteobacteria (5.0 ± 1.4%), and Desulfobacteria (4.6 ± 1.8%). The average relative abundance of the remaining classes did not surpass 4% (Figure 4).

The structure of the bacterial community exhibits distinct horizontal and vertical distribution patterns. The relative abundance of specific bacterial phyla, such as Pseudomonadota (including Gammaproteobacteria and Alphaproteobacteria), Thermodesulfobacteriota (primarily comprising Desulfobacteria, Syntrophobacteria, and Desulfobulbia), and Acidobacteriota, showed increases with distance from the shore, extending from nearshore to offshore environments (Figure 4). In contrast, the relative abundance of several bacterial taxa, such as Chloroflexota (e.g., Anaerolineae and Dehalococcoidia), Actinomycetota, and Bacillota, decreased offshore. Additionally, some bacterial taxa like Chloroflexota and Bacillota were more prevalent in deeper sediments, while the relative abundance of Gammaproteobacteria diminished with increasing depth (Figure 4).

## 4. Discussion

### 4.1. Source Identification of IPL- and CL-brGDGTs

The study area is located in the nearshore zone of the ECS. The δ^13^C values (Figure 2c) indicate that the sedimentary organic matter is derived from both terrestrial inputs and marine autochthonous sources, consistent with previous findings [62,80,81]. In this section, we discuss the sources of IPL- and CL-brGDGTs in multiple sediment cores from the ECS. IPL-brGDGTs were primarily derived from in situ production, whereas CL-brGDGTs were significantly influenced by marine autochthonous inputs.

#### 4.1.1. IPL-brGDGTs Primarily Produced In Situ

In the sediment cores of the study area, the TOC-normalized IPL-brGDGT concentration increased substantially offshore (Figure 3). When compared with the mid-lower Yangtze River basin soils (IPL/CL_br_: 0.04) [26,82] and the YRE surface sediments (IPL/CL_br_: 0.06) [23], the IPL/CL_br_ ratio in this study was remarkably higher (0.12–0.49; Figure 3, Table S1). Given that IPL-brGDGTs are more labile than CL-brGDGTs, they are unlikely to be selectively retained amid the significant degradation in the YRE [42]. This suggests that IPL-brGDGTs in sediments of the study area were predominantly produced in situ, with a higher turnover rate (i.e., production/degradation ratio)—consistent with previous findings in ECS surface sediments [23,49]. Elevated IPL/CL_br_ values were mainly observed at site A2, presumably due to the increased bacterial abundance in muddy zone sediments [83].

Furthermore, the MAT (12.6–17.2 °C, average 14.8 ± 1.0 °C) and soil pH (6.66–7.44, average 7.01 ± 0.17) derived from IPL-brGDGT-based ${{MBT}^{\prime }}_{5Me}$ and ${CBT}_{5Me}$ (Table S1) do not match those in the mid-lower Yangtze River basin (MAT: 16–18 °C [84]; pH < 6.5 [85]), further indicating that IPL-brGDGTs were not significantly influenced by terrestrial input. However, according to the calibration formula (BWT = 59.5 × ${{MBT}^{\prime }}_{5Me}$ − 23.7) [20], the bottom water temperature (BWT) calculated from IPL-brGDGTs in surface sediments at each site ranged from 18.9 to 22.1 °C. This is similar to the measured BWT during sampling (18.4–21.5 °C), suggesting that the IPL-brGDGTs in the surface sediments were likely synthesized in situ recently by bottom marine bacteria. A similar phenomenon was observed in the study of surface sediments from the ECS [49]. In fact, high turnover rates of IPL-brGDGTs have been reported in lacustrine and marine sediments, with their distribution patterns influenced by ambient conditions during sampling [23,25,26,49]. Given the relatively low sedimentation rate in the study area (<2 cm/year [86]), it is reasonable to postulate that IPL-brGDGTs in the sediment cores (aged at least 15 years) were predominantly synthesized in situ by living bacteria, rather than through the deposition of bottom-water column material.

**Figure 5:**
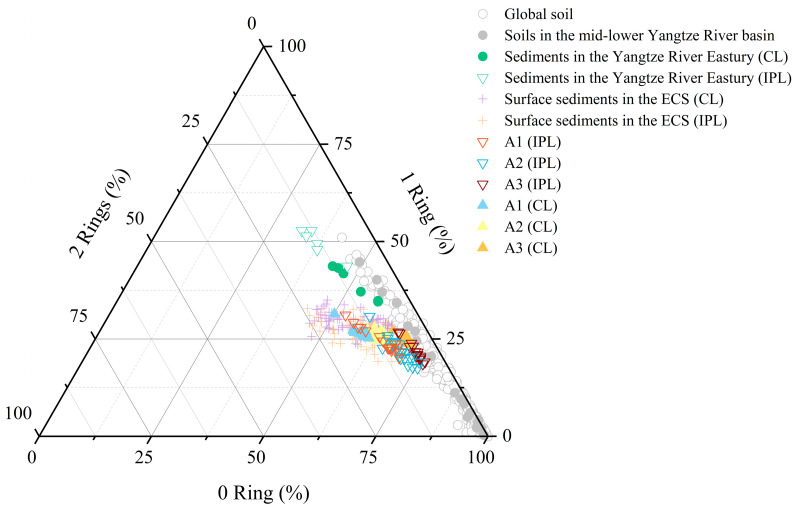
Ternary diagram illustrating the relative abundances (%) of brGDGTs with 0, 1, and 2 cyclopentane moieties across several environmental samples. The dataset comprises global soils [15] (grey open circles), soils from the mid-lower Yangtze River basin [87,88,89,90] (grey solid circles), surface sediments from the Yangtze River Estuary [49] (green solid circles for CL-brGDGTs, green inverted triangles for IPL-brGDGTs), surface sediments from the East China Sea [23,35,49] (purple crosses for CL-brGDGTs, orange crosses for IPL-brGDGTs), and core sediment samples from this study (open inverted triangles for IPL-brGDGTs and solid triangles for CL-brGDGTs).

**Figure 6:**
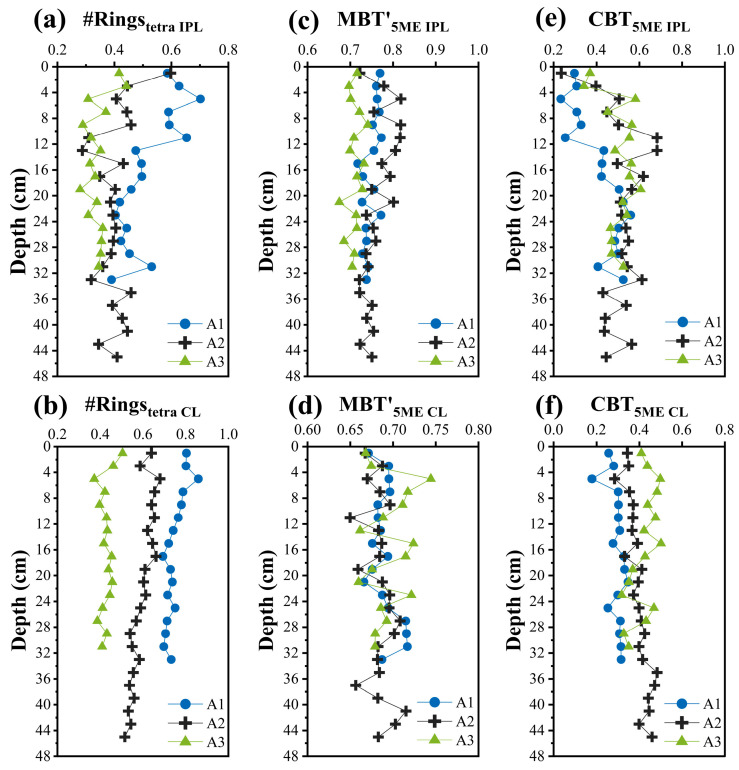
Vertical profiles of brGDGTs-derived indices in sediment cores from sites A1 (blue circles), A2 (black crosses), and A3 (green triangles): (**a**) ${\#Rings}_{tetra}$ calculated from IPL-brGDGTs, (**b**) ${\#Rings}_{tetra}$ calculated from CL-brGDGTs; (**c**) ${{MBT}^{\prime }}_{5Me}$ calculated from IPL-brGDGTs, (**d**) ${{MBT}^{\prime }}_{5Me}$ calculated from CL-brGDGTs; (**e**) ${CBT}_{5Me}$ calculated from IPL-brGDGTs, (**f**) ${CBT}_{5Me}$ calculated from CL-brGDGTs.

IPL-brGDGTs exhibited distinct spatial distribution patterns: the relative abundance of those with two cyclopentane rings increased offshore, whereas that of ring-free IPL-brGDGTs showed a slight decrease offshore (Figure 5). These differences resulted in a general upward tendency in the IPL-brGDGT-derived ${\#Rings}_{tetra}$ values, which ascended from 0.34 ± 0.04 at the nearshore site to 0.51 ± 0.09 offshore (Figure 6a). Similar findings have been reported in surface sediments from the Svalbard fjord [25] and the ECS [23,49], which were suggested to result from environmental gradients (e.g., salinity, pH) and/or shifts in brGDGT-producing bacterial communities. Cao et al. [23] suggested that the distribution of IPL-brGDGTs in the surface sediments of the ECS was significantly affected by seawater pH: as pH increased offshore, the cyclization degree of IPL-brGDGTs rose, consistent with trends observed in soils [14,15]. Nevertheless, to date, no studies have assessed the impact of changes in bacterial community structure on the distribution of IPL-brGDGTs in sediments.

**Figure 7:**
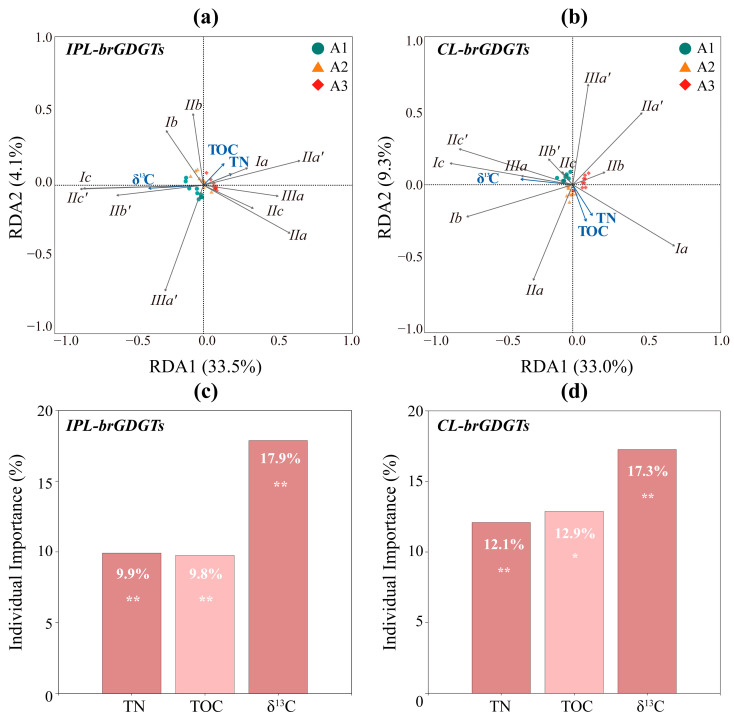
Partial RDA analysis illustrating the correlations between total organic compositions (TOC, TN, δ^13^C; blue arrows) and the fractional abundances of IPL-brGDGTs (**a**) and CL-brGDGTs (**b**). The individual importance of each organic composition (TOC, TN, δ^13^C) explaining the variation in the IPL-brGDGTs (**c**) and CL-brGDGTs (**d**) distributions was assessed by hierarchical partitioning analysis. The dataset used for partial RDA analysis comprises sediment samples from site A1 (*n* = 9; green circles), site A2 (*n* = 12; yellow triangles), and site A3 (*n* = 9; red rhombuses). Asterisks show the significance level: * *p* < 0.05; ** *p* < 0.01.

Partial RDA analysis revealed that the δ^13^C of sedimentary organic matter, along with TOC and TN contents, explained 37.6% of the variability in the distribution of IPL-brGDGTs across the two axes. The samples from the three sites formed distinct clusters, indicating that these variables predominantly accounted for the horizontal spatial variation in IPL-brGDGTs from terrestrial to marine environments (Figure 7a). Specifically, δ^13^C alone accounted for 17.9% of the variation (F = 9.66, *p* < 0.01, Figure 7c), while TOC and TN accounted for 9.8% (F = 2.64, *p* < 0.01) and 9.9% (F = 4.35, *p* < 0.05), respectively (Figure 7c). Notably, δ^13^C exhibited a positive correlation with Ic, IIc’, and IIb’ (Figure 7c), suggesting that offshore areas with higher δ^13^C values are more favourable for the synthesis of these cyclized and 6-methyl brGDGTs, which is consistent with previous findings [22,35,67]. Meanwhile, TOC and TN were positively correlated with Ia and IIa’ (Figure 7a), indicating that in nearshore environments with high TOC and TN contents, brGDGTs are mainly composed of Ia and IIa’. Similar phenomena have also been observed in earlier studies on ECS surface sediments [35] and Seine River Estuary SPM [91]. It is considered that the primary producers of brGDGTs are heterotrophic bacteria [92,93], which participate in the biogeochemical cycling of carbon and nitrogen through the transformation of organic matter [94,95,96].

In summary, IPL-brGDGTs in the sediment cores of the study area primarily originated from in situ biosynthesis. Their distribution exhibited an increasing cyclization degree offshore, influenced by the source and content of total organic matter (TOM).

#### 4.1.2. CL-brGDGTs Significantly Impacted by the Autochthonous Contribution

CL-brGDGTs in the surface sediments of the ECS are considered to be affected by both terrestrial inputs [44,46,48] and marine sources, such as planktonic [22] and benthic bacterial production [35]. As shown in Figure 5, the distribution of CL-brGDGTs in this study differs from that of soils in the mid-lower Yangtze River basin [87,88,89,90] and surface sediments from the YRE [23]. The differences are more pronounced seaward (Figure 5), indicating a significant influence of marine-autochthonous input. It has been generally accepted that soil-derived brGDGTs demonstrate a lower degree of cyclization, while marine-autochthonous ones show a higher cyclization degree [25,67,97,98]. The average ${\#Rings}_{tetra}$ index for soil brGDGTs in the mid-lower Yangtze River basin is 0.25 ± 0.20 [87,88,89,90], and can reach up to 1.06 in the sediments of the outer ECS shelf [22]. In this study, the CL-brGDGT-derived ${\#Rings}_{tetra}$ values increase from nearshore areas (0.43 ± 0.03) to offshore (0.75 ± 0.05) regions (Figure 6), indicating a significant contribution of autochthonous CL-brGDGTs in offshore sediments [44,45,46,49]. Notably, CL-brGDGTs exhibit similar spatial distribution patterns to IPL-brGDGTs (Figure 5). Therefore, the lower cyclization degree of CL-brGDGTs in nearshore sediments might be a characteristic of in situ bacterial membrane lipids [23,49], further suggesting that the CL-brGDGTs in the study area are likely to be significantly influenced by marine in situ production.

Moreover, the ${{MBT}^{\prime }}_{5Me}$ and ${CBT}_{5Me}$ values obtained from CL-brGDGTs also failed to replicate the MAT (16–18 °C [84]) and soil pH (<0.65 [85]) of the mid-lower Yangtze River basin. The reconstructed temperatures were too low (11.9–14.8 °C, with a mean of 13.1 ± 0.6 °C; Table S1), while the pH values were too high (6.97–7.53, with a mean of 7.20 ± 0.12; Table S1). These discrepancies support the significant contribution of marine in situ production to CL-brGDGTs in the study area.

Similarly, the δ^13^C, TOC, and TN contents of sedimentary organic matter explained the distribution patterns of CL-brGDGTs. Their combined explanatory power across the two axes exceeded that for IPL-brGDGTs, reaching 42.3%. Specifically, δ^13^C alone explained 17.3% of the variation (F = 12.16, *p* < 0.01), which is comparable to its explanatory capacity for IPL-brGDGTs. TOC and TN contents explained 12.9% (F = 2.47, *p* < 0.01) and 12.1% (F = 4.92, *p* < 0.05), respectively, indicating a greater influence on CL-brGDGTs than on IPL-brGDGTs (Figure 7d). δ^13^C was primarily positively correlated with Ic, IIc′, and IIIa in CL-brGDGTs (Figure 7b), suggesting that these brGDGTs were more enriched in areas where sedimentary organic matter was predominantly of marine origin [67,99]. Meanwhile, TOC and TN contents were mainly positively correlated with Ia and IIa in CL-brGDGTs (Figure 7b), which were more abundant in high-productivity nearshore environments. The similarity in the spatial distribution patterns of CL- and IPL-brGDGTs further supports previous findings indicating that the contribution of marine autochthonous sources to CL-brGDGTs in the coastal ECS may have been underestimated [23,49].

In contrast to IPL-brGDGTs, the cyclization indices (i.e., ${\#Rings}_{tetra}$ and ${CBT}_{5Me}$) derived from CL-brGDGTs exhibited small variations across the depth profiles of the three sediment cores (Figure 6b,f). At varying depths, the ${\#Rings}_{tetra}$ values retained the similar spatial disparities found in surface sediments. These indicate that CL-brGDGTs have relatively stable sources and are less affected by vertical environmental changes and sediment disturbances.

### 4.2. The Relationship Between Bacterial Community Diversity and Variations in BrGDGT Distribution

Analogous to brGDGTs, the composition of bacterial communities in the sediment cores of the research area exhibited spatial variation along the nearshore-to-offshore continuum of benthic habitats. In offshore areas, the relative abundance of bacterial taxa such as Alphaproteobacteria, Thermodesulfobacteriota, and Acidobacteriota increased, while those of Chloroflexota, Actinomycetota, and Bacillota declined (Figure 4).

#### 4.2.1. The Potential Biological Producers of BrGDGTs

**Figure 8:**
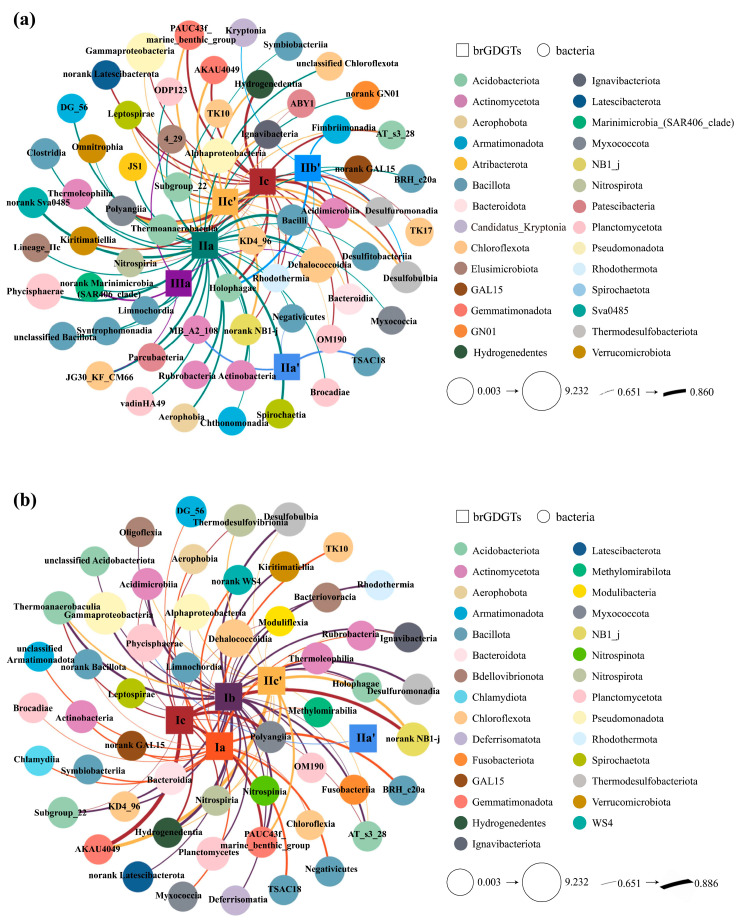
The co-occurrence network depicts Spearman’s rank correlations between bacterial community (at class levels) and IPL-brGDGTs (**a**), as well as CL-brGDGTs (**b**), based on relative abundances (*r* > 0.65, *p* < 0.05). Nodes represent bacterial taxa (circles) and brGDGTs (squares); the degree of correlation is indicated by the width of the edge, and the relative abundance of bacterial taxa is shown by the size of the node. Taxonomic classifications are represented through colour coding according to phylum.

A co-occurrence network was established via Spearman correlation to elucidate the potential association between bacterial taxa and the distribution patterns of brGDGTs. Consequently, bacterial taxa at the class level that showed strong correlations with brGDGTs (*r* > 0.65, *p* < 0.05) were uncovered (Figure 8). 63 bacterial groups were recognized as being related to six chemicals in IPL-brGDGTs (Ic, IIc′, IIb′, IIa, IIa′, and IIIa) (Figure 8a). Moreover, 55 bacterial groups were found to be associated with five chemicals in CL-brGDGTs (Ic, Ib, Ia, IIc′, and IIa′) (Figure 8b). These bacterial taxa mainly belong to Pseudomonadota (Gammaproteobacteria, Alphaproteobacteria), Chloroflexota, Acidobacteriota, Bacillota, and Planctomycetota.

Comparisons with previously established co-occurrence networks in ECS SPM [22] and surface sediments [35] showed consistency with the results of this study. The bacterial taxa linked to brGDGTs, including Gammaproteobacteria, Alphaproteobacteria, Chloroflexota (comprising Dehalococcoidia and Anaerolineae), Phycisphaerae, and Planctomycetes from Planctomycetota, were similarly identified in those investigations. These bacterial taxa have been reported to harbour homologs of key enzymes involved in GDGT biosynthetic pathways [32,33,34], including the tetraether synthetase (Tes) responsible for isoprenoid coupling [32], along with the Mss and Ger enzymes that mediate fatty acid coupling and ether bond formation [31]. This suggests that these bacteria are likely the main potential producers of brGDGTs across the nearshore-to-offshore benthic continuum in the ECS.

To further clarify potential links between bacterial taxa and brGDGT distribution patterns across the inshore–offshore gradient, a co-occurrence network of bacterial communities and brGDGTs was constructed for each sampling site based on Spearman correlations (Figure S1). The results revealed subtle differences in the primary potential brGDGT producers among sites.

At the offshore site (A1), the bacterial classes significantly correlated with IPL- and CL-brGDGTs were primarily Anaerolineae, Gammaproteobacteria, Desulfobacteria, and Phycisphaerae (Figure S1a,b). This could be attributed to the seasonal hypoxia phenomenon (July–September) occurring at the site [100]. Anaerolineae, a strictly anaerobic chemoheterotrophic group, predominates in organic-rich marine sediments under hypoxic or microoxic conditions, where it may decompose complex organic substances [101,102]. Gammaproteobacteria, composed of facultative anaerobic chemolithotrophic bacteria, is mainly situated in YRE sediments [103]. With the expansion of coastal hypoxic zones due to global warming, the expression of narG genes in Gammaproteobacteria increases significantly under low-oxygen conditions [104]. Desulfobacteria are obligate anaerobic chemolithotrophs [105], and their rapid sulfur reduction can enhance the deposition of organic matter in modern hypoxic zone sediments [106]. Phycisphaerae, a strictly anaerobic chemoheterotrophic group [107,108], dominates hypoxic and suboxic zone sediments, contributing to nitrogen cycling and organic carbon degradation [109].

At the coastal site (A3), the bacterial classes associated with brGDGTs primarily included Gammaproteobacteria, Alphaproteobacteria, Actinobacteria, Dehalococcoidia, and Bacilli (Figure S1e,f). This may relate to site A3’s location within the dissolved organic carbon sink region near the YRE [110]. Alphaproteobacteria, an aerobic chemoheterotrophic group, are enriched in carbon metabolism genes in ECS nearshore sediments and play a vital role in organic matter decomposition [111]. Actinobacteria are predominantly strict aerobic chemolithotrophs in the nearshore ECS, with their distribution tightly linked to the inputs of terrestrial organic matter [112]. Actinobacteria participate in the carbon cycle by degrading macromolecules such as cellulose and chitin, thereby providing nutritional sources for other organisms. Additionally, they help maintain the balance of microbial communities through the production of antibiotics [113]. Studies suggest that the organic-rich nearshore zones are key habitats for the chemolithotrophic Dehalococcoidia group. Although Dehalococcoidia does not directly break down complex organic waste, its dehalogenation processes can indirectly facilitate carbon cycling [102]. In the nearshore ECS, Bacilli are predominantly aerobic chemoheterotrophs that degrade phytoplankton detritus and terrestrial organic materials, transforming particulate organic carbon into dissolved organic carbon and CO_2_ [114].

In summary, at the class level, chemolithotrophic bacteria, including Gammaproteobacteria and Dehalococcoidia, along with chemoheterotrophic bacteria such as Alphaproteobacteria, Bacilli, and Actinobacteria, were identified as crucial potential brGDGT producers in the nearshore ECS. Conversely, chemoheterotrophs capable of thriving in anoxic environments, like Anaerolineae and Phycisphaerae, together with facultative anaerobic chemolithotrophs such as Gammaproteobacteria and Desulfobacteria, were predominant in the offshore ECS.

#### 4.2.2. Influence of Bacterial Community Diversity on the Distribution of BrGDGTs

Furthermore, we performed a cluster analysis on the bacterial communities that showed significant positive correlations with both IPL- and CL-brGDGTs. The results revealed that the distribution patterns of bacterial community composition across all sites changed remarkably at a depth of 17 cm (Figure S2). Sediment samples from depths above 17 cm tended to cluster, displaying a similar community composition and abundance. Conversely, samples from depths below 17 cm had greater variability, demonstrating significant disparities in community composition and abundance. Likewise, IPL-brGDGT-derived ${\#Rings}_{tetra}$ and ${CBT}_{5Me}$ above 17 cm exhibited notable variations among sites, with increased volatility. However, the disparities in cyclization levels across the three sites diminished markedly, and the variations became more stable at the depths below 17 cm (Figure 6a,e). An independent samples t-test indicated significant differences in ${\#Rings}_{tetra}$ (or ${CBT}_{5Me}$) values between samples at depths above and below 17 cm (*p* < 0.05), and the former exhibited greater variance (Levene’s test *p* < 0.001).

Previous studies have indicated that near-surface sediments are highly vulnerable to physical disturbances such as waves, tides, and storms [115]. In the muddy areas along the Zhejiang-Fujian coastline, the remobilized muddy sediments, mainly affected by physical processes, generally have a depth range of 10–30 cm, and in some areas, it can reach up to 50 cm [116,117]. This has resulted in significant and intricate fluctuations in environmental parameters such as redox potential, pH, and nutrient contents within the near-surface sediments [116]. These changes can fundamentally explain the observed depth-related differences in bacterial community composition and IPL-brGDGTs distribution at a depth of 17 cm. Qiao et al. investigated the diversity of sediment bacterial communities in the mud regions of the ECS and observed similar depth-related variations [83]. Specific hydrodynamic conditions (including the TWC, tides, etc.), oxygen concentrations, and the characteristics of organic matter may be key factors influencing the depth-dependent distribution variations of benthic bacterial communities.

Due to the heterogeneity of sedimentary environments, benthic bacterial communities may exhibit distinct physiological and ecological mechanisms across different sediment depths. In this study, turbulent conditions above 17 cm and stable conditions below likely drove divergent physiological adaptations and ecological strategies among bacterial communities, consequently leading to variations in IPL-brGDGT cyclization patterns.

### 4.3. Assessment of the Effects of Bacterial Community and the Composition of TOM on brGDGT Distribution

Since the distribution patterns of brGDGTs are affected by both bacterial community composition (Figure 8) and TOM composition (Figure 7), VPA was employed to assess the extent to which these two factors influence brGDGT distribution. This approach can quantify the distinct variation (i.e., marginal impact) and the shared variation (i.e., common effect) among predictor factors or their combinations.

**Figure 9:**
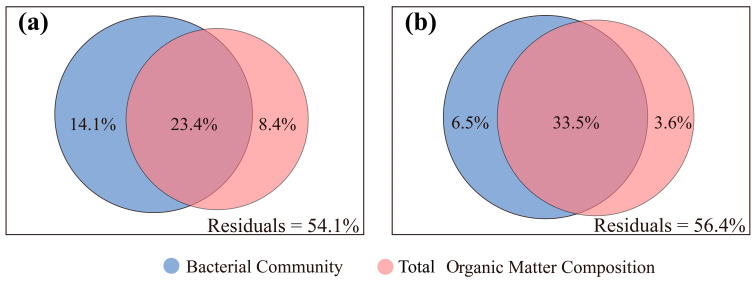
Venn diagram illustrating the results of variation partitioning analysis (VPA), depicting the respective contributions of significantly correlated bacterial community structures (at the phylum level) and total organic matter composition to the distributions of IPL-brGDGTs (**a**) and CL-brGDGTs (**b**). The VPA was carried out with parameters: α = 0.05 (significance level), test power = 0.8 (ensuring sufficient power to detect meaningful effects), 1000 permutations (for robust *p*-value estimation), and 95% CIs (to quantify the uncertainty of the results).

The results of VPA demonstrated that the distribution of brGDGTs is predominantly affected by the bacterial community, with the composition of TOM having a secondary effect. Together, the composition of the bacterial community and TOM accounted for 45.9% of the variation in the distribution of IPL-brGDGTs. More specifically, the bacterial community composition alone contributed 14.1%, the TOM composition independently contributed 8.4%, and the combined effect of both factors accounted for 23.4% of the variation (Figure 9a). Concerning the distribution of CL-brGDGTs, the bacterial community composition and TOM composition collectively explained 43.6% of the variation, with individual contributions of 6.5% and 3.6%, respectively, and an overlapping contribution of 33.5% (Figure 9b). Clearly, the cumulative influence of the bacterial community composition and TOM composition on IPL-brGDGTs is greater than that on CL-brGDGTs (45.9% > 43.6%), suggesting a stronger correlation between IPL-brGDGTs and the bacterial community composition as well as TOM in marine sediments.

It has been suggested that the composition of sedimentary TOM significantly influences the distribution of the benthic bacterial community structure, potentially reflecting the selective substrate preferences of heterotrophic bacteria [35,83,118]. This could explain why their combined effect on brGDGT distribution far outweighed individual contributions (Figure 9). Individually, the changes in bacterial community structure accounted for a significantly larger proportion of the variation in brGDGT distribution than TOM composition did, highlighting the former’s greater role in driving such shifts. This discovery is consistent with earlier findings that the distribution pattern of brGDGTs is significantly influenced by benthic environmental and chemical factors, as well as bacterial communities [35]. Compared with CL-brGDGTs, the independent impact of the bacterial communities on the distribution of IPL-brGDGTs is more pronounced (14.1% vs. 6.5%) (Figure 9), indicating that the main source of IPL-brGDGTs is likely in situ synthesis by benthic bacteria.

## 5. Conclusions

Based on the analysis of sediment cores collected along a nearshore-to-offshore transect in the ECS, this study presents the following findings: (1) In sediment cores, IPL-brGDGTs mainly originate from in situ bacterial synthesis. Conversely, CL-brGDGTs derive from an integrated lipid reservoir, which includes terrestrial inputs and products of marine planktonic and benthic bacteria. Marine autochthonous bacteria contribute significantly to the sedimentary CL-brGDGTs pool, particularly in offshore environments. (2) At the class level, potential producers of brGDGTs in nearshore environments primarily include chemolithoautotrophic bacteria such as Gammaproteobacteria and Dehalococcoidia, as well as chemoheterotrophic bacteria such as Alphaproteobacteria, Bacilli, and Actinobacteria. In offshore regions, the main potential producers of brGDGTs are predominantly anoxic-adapted chemolithoautotrophic bacteria, such as Anaerolineae and Phycisphaerae, along with facultative anaerobic chemoautotrophic bacteria, including Gammaproteobacteria and Desulfobacteria. (3) A distinct depth-dependent differentiation in microbial composition and IPL-brGDGT distribution is observed above and below 17 cm, which may be attributed to the greater susceptibility of near-surface sediments to physical disturbances such as wave action, tidal forces, and storm events. (4) In the nearshore-to-offshore sedimentary ecosystems of the ECS, the distribution of brGDGTs is primarily governed by the composition of the bacterial community. In contrast, the influence of TOM composition appears relatively minor.

These findings improve our understanding of marine brGDGT production and the regulatory mechanisms that control its distribution in marginal seas. Furthermore, they emphasize the importance of considering physical disturbance effects when interpreting sedimentary brGDGT records for paleoenvironmental reconstructions in coastal marine settings.

## Figures and Tables

**Table 1:** Information of sediment cores.

Site	Longitude (°E)	Latitude (°N)	Water Depth (m)	Length (cm)
A1	123.11	29.84	67.3	34
A2	122.89	29.91	55.5	46
A3	122.64	30.00	36.9	31

## Data Availability

Data accessible in a publicly accessible repository; specifically, the original data presented in the study are openly available in the NCBI database under BioProject ID PRJNA1283994.

## Supplementary Materials

The following supporting information can be downloaded at: https://www.mdpi.com/article/10.3390/biology14081077/s1, Figure S1: The co-occurrence network illustrating the Spearman’s rank correlations between bacterial communities (at class levels) and IPL-brGDGTs in sediment cores from sites. Figure S2: Hierarchical clustering of sediment samples based on bacterial taxa (at the class level), Table S1: The contents and distribution of brGDGTs in the sediment samples of this study.

## Abbreviations

The following abbreviations are used in this manuscript:

brGDGTs	Branched glycerol dialkyl glycerol tetraethers
IPL-brGDGTs	Intact polar lipid-derived brGDGT
CL-brGDGTs	Core brGDGTs
ECS	East China Sea
VPA	Variance partitioning analysis
IPLs	Intact polar lipids
CLs	core lipids
SPM	suspended particulate matter
SST	sea surface temperature (SST)
YDW	Yangtze River Diluted Water
ZFCC	Zhejiang-Fujian Coastal Current
TWC	Taiwan Warm Current
YRZ	Yangtze River Estuary
NKBC	Nearshore Kuroshio Branch Current
TOC	total organic carbon
TN	total nitrogen
TOM	total organic matter
SDs	standard deviations
MeOH	methanol
DCM	dichloromethane
TLE	Total lipid extract
OTUs	Operational taxonomic units
Partial RDA	Partial redundancy analysis
BWT	Bottom water temperature
