# Supplementary material for: Distribution and Variation Characteristics of Branched Glycerol Dialkyl Glycerol Tetraethers (BrGDGTs) in Sediment Cores Along the Nearshore-to-Offshore Gradient of the East China Sea and Their Correlation with Microbial Community Diversity
Source: Biology (Basel). 2025 Aug 18;14(8):1077. doi: 10.3390/biology14081077 (PMC12383918; doi:10.3390/biology14081077)
Supplement: Supplementary file 1 [file biology-14-01077-s001.zip › Supplementary Materials_Figure/Supplementary Figures.pdf]

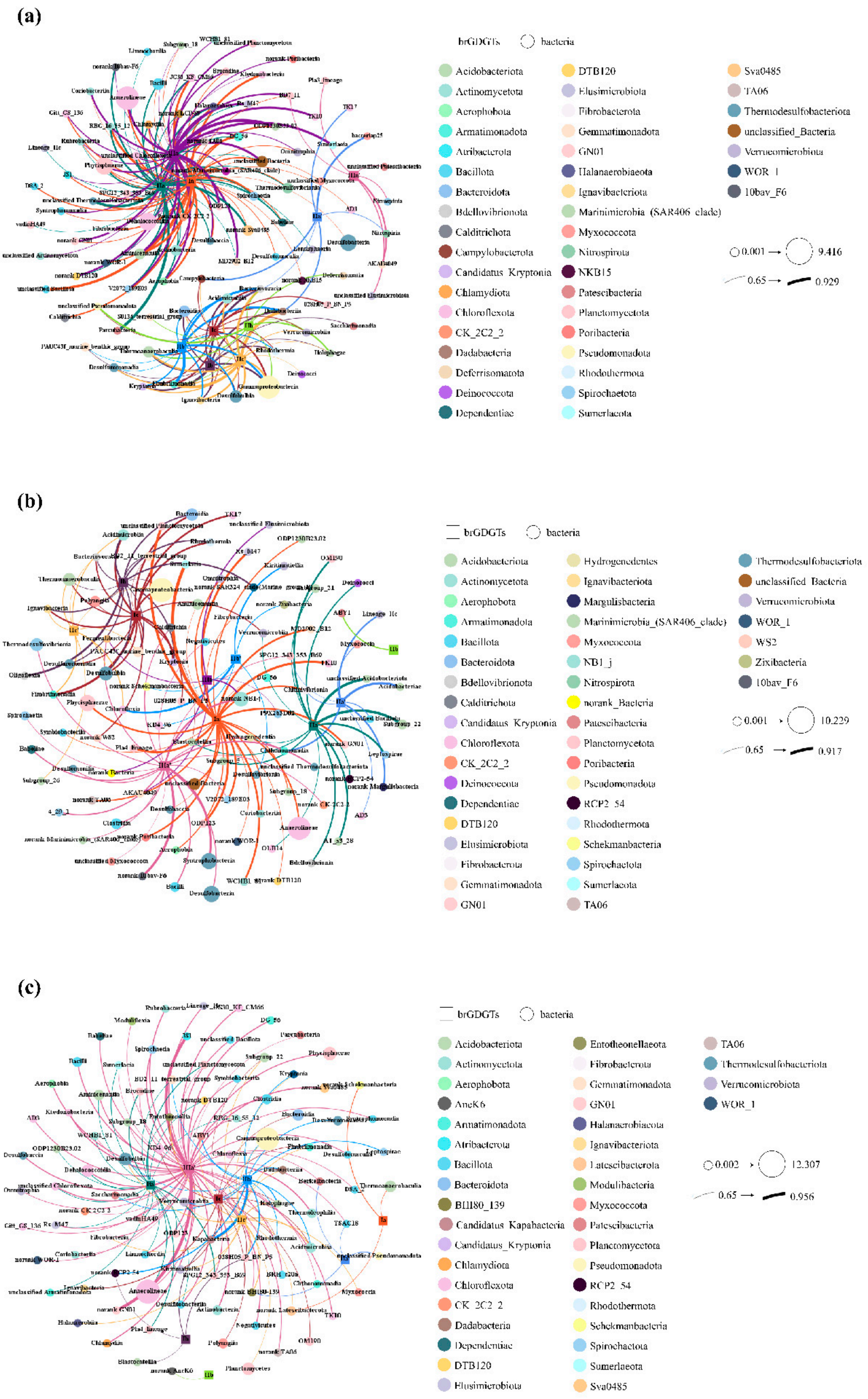

(a)
brGDGTs
bacteria
Acidobacteriota
Actinomycetota
Aerophobota
Armatimonadota
Atribacteriota
Bacillota
Bacteroidota
Bdellovibrionota
Calditrichota
Campylobacterota
Candidatus_Kryptonia
Chlamydiota
Chloroflexota
CK_2C2_2
Dadabacteria
Deferrisomatota
Deinococcota
Dependentiae
DTB120
Elusimicrobiota
Fibrobacterota
Gemmatimonadota
GN01
Halanaerobiaeota
Ignavibacteriota
Marinimicrobia_(SAR406_clade)
Myxococcota
Nitrospirota
NKB15
Patescibacteria
Planctomycetota
Poribacteria
Pseudomonadota
Rhodothermota
Spirochaetota
Sumerlaeota
Sva0485
TA06
Thermodesulfobacteriota
unclassified_Bacteria
Verrucomicrobiota
WOR_1
10bav_F6
0.001
9.416
0.65
0.929
(b)
brGDGTs
bacteria
Acidobacteriota
Actinomycetota
Aerophobota
Armatimonadota
Bacillota
Bacteroidota
Bdellovibrionota
Calditrichota
Candidatus_Kryptonia
Chloroflexota
CK_2C2_2
Deinococcota
Dependentiae
DTB120
Elusimicrobiota
Fibrobacterota
Gemmatimonadota
GN01
Hydrogenedentes
Ignavibacteriota
Margulisbacteria
Marinimicrobia_(SAR406_clade)
Myxococcota
NB1_j
Nitrospirota
norank_Bacteria
Patescibacteria
Planctomycetota
Poribacteria
Pseudomonadota
RCP2_54
Rhodothermota
Schekmanbacteria
Spirochaetota
Sumerlaeota
TA06
Thermodesulfobacteriota
unclassified_Bacteria
Verrucomicrobiota
WOR_1
WS2
Zixibacteria
10bav_F6
0.001
10.229
0.65
0.917
(c)
brGDGTs
bacteria
Acidobacteriota
Actinomycetota
Aerophobota
AncK6
Armatimonadota
Atribacteriota
Bacillota
Bacteroidota
BHI80_139
Candidatus_Kapabacteria
Candidatus_Kryptonia
Chlamydiota
Chloroflexota
CK_2C2_2
Dadabacteria
Dependentiae
DTB120
Elusimicrobiota
Entotheonellaeota
Fibrobacterota
Gemmatimonadota
GN01
Halanaerobiaeota
Ignavibacteriota
Latescibacterota
Modulibacteria
Myxococcota
Patescibacteria
Planctomycetota
Pseudomonadota
RCP2_54
Rhodothermota
Schekmanbacteria
Spirochaetota
Sumerlaeota
Sva0485
TA06
Thermodesulfobacteriota
Verrucomicrobiota
WOR_1
0.002
12.307
0.65
0.956

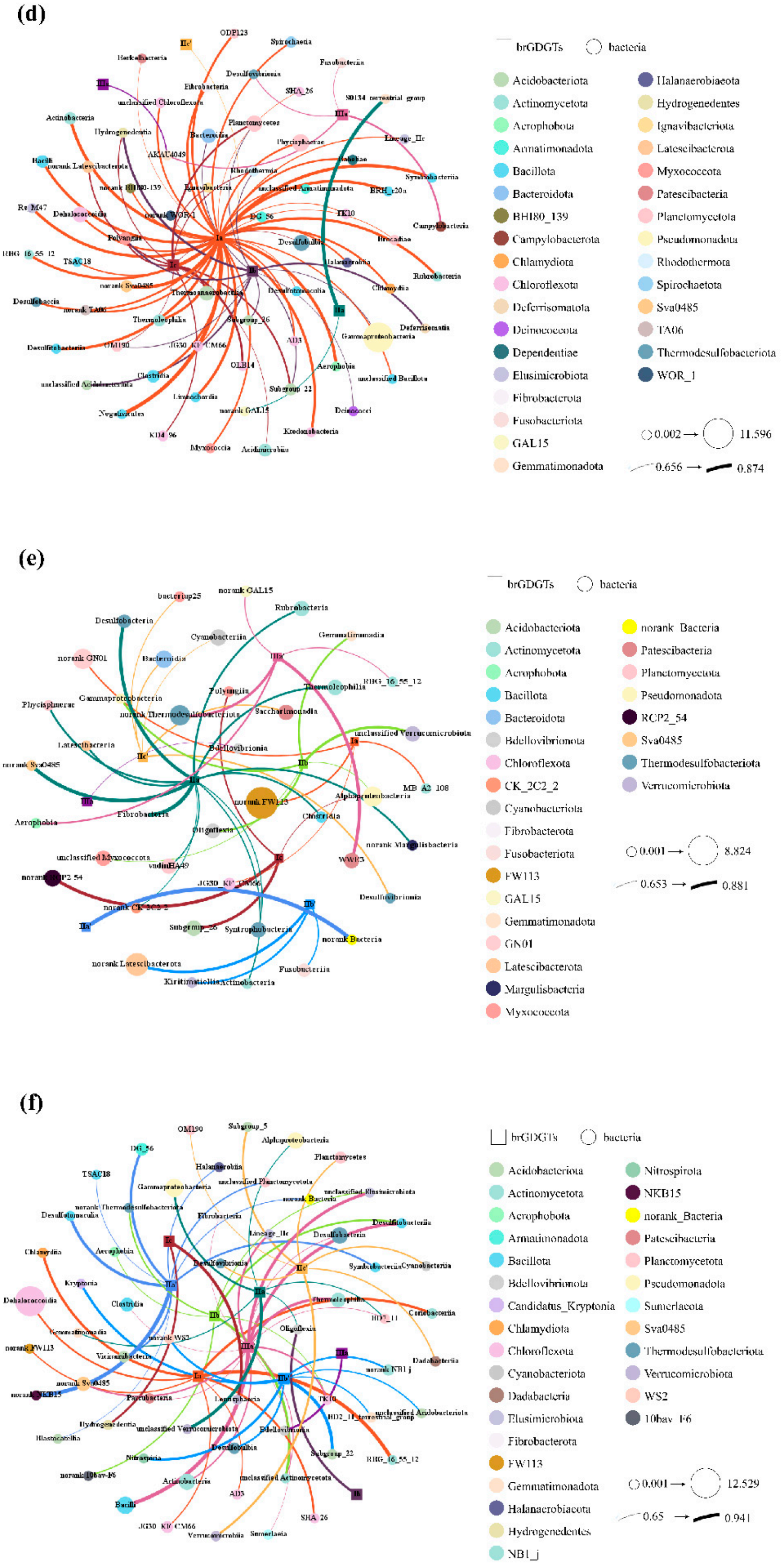
(d)
brGDGTs
bacteria
Acidobacteriota
Actinomycetota
Aerophobota
Armatimonadota
Bacillota
Bacteroidota
BHI80_139
Campylobacterota
Chlamydiota
Chloroflexota
Deferrisomatota
Deinococcota
Dependentiae
Elusimicrobiota
Fibrobacterota
Fusobacteriota
GAL15
Gemmatimonadota
Halanaerobiaeota
Hydrogenedentes
Ignavibacteriota
Latescibacterota
Myxococcota
Patescibacteria
Planctomycetota
Pseudomonadota
Rhodothermota
Spirochaetota
Sva0485
TA06
Thermodesulfobacteriota
WOR_1
0.002 → 11.596
0.656 → 0.874
(e)
brGDGTs
bacteria
Acidobacteriota
Actinomycetota
Aerophobota
Bacillota
Bacteroidota
Bdellovibrionota
Chloroflexota
CK_2C2_2
Cyanobacteriota
Fibrobacterota
Fusobacteriota
FW113
GAL15
Gemmatimonadota
GN01
Latescibacterota
Margulisbacteria
Myxococcota
norank_Bacteria
Patescibacteria
Planctomycetota
Pseudomonadota
RCP2_54
Sva0485
Thermodesulfobacteriota
Verrucomicrobiota
0.001 → 8.824
0.653 → 0.881
(f)
brGDGTs
bacteria
Acidobacteriota
Actinomycetota
Aerophobota
Armatimonadota
Bacillota
Bdellovibrionota
Candidatus_Kryptonia
Chlamydiota
Chloroflexota
Cyanobacteriota
Dadabacteria
Elusimicrobiota
Fibrobacterota
FW113
Gemmatimonadota
Halanaerobiaeota
Hydrogenedentes
NB1_j
Nitrospirota
NKB15
norank_Bacteria
Patescibacteria
Planctomycetota
Pseudomonadota
Sumerlaeota
Sva0485
Thermodesulfobacteriota
Verrucomicrobiota
WS2
10bav_F6
0.001 → 12.529
0.65 → 0.941

Figure S1. The co-occurrence network illustrating the Spearman's rank correlations between bacterial communities (at class levels) and IPL-brGDGTs in sediment cores from sites A1 **(a)**, A2 **(c)** and A3 **(e)** and CL-brGDGTs in sediment cores from sites A1 **(b)**, A2 **(d)** and A3 **(f)** based on relative abundances ($r > 0.65$, $p < 0.05$).

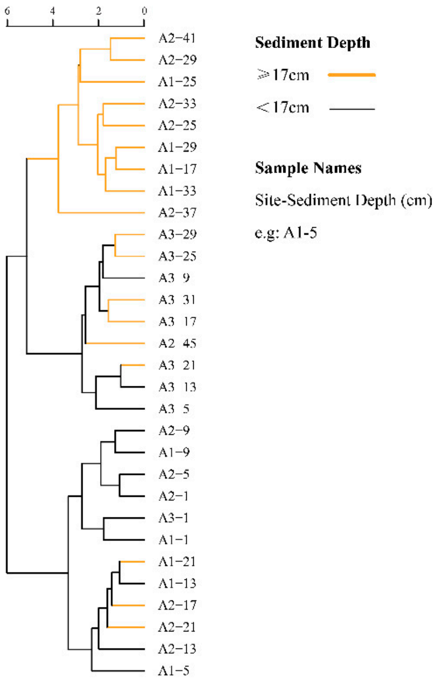

Figure S2. Hierarchical clustering of sediment samples based on bacterial taxa (at the class level) significantly correlated with brGDGTs. Orange lines represent sediment samples from depths ≥ 17 cm, while black lines represent sediment samples from depths < 17 cm. The dendrogram was constructed based on microbial amplicon sequencing data (16S rRNA gene) from sediment samples. Sample names follow the format [site] - [sediment depth] (e.g., A1-5 indicates 5 cm depth sediment from site A1). Only bacterial taxa (at class levels) significantly positively correlated ($p < 0.05$) with both IPL-brGDGTs and CL-brGDGTs were included. Relative abundances were Z-score standardized prior to clustering. Euclidean distance and complete linkage were used to construct the dendrogram. Samples exhibiting closely related branch lengths demonstrate higher similarity in microbial community composition.
